# From Concept to Clinical Product: A Brief History of the Novel *Shigella* Invaplex Vaccine’s Refinement and Evolution

**DOI:** 10.3390/vaccines10040548

**Published:** 2022-04-01

**Authors:** K. Ross Turbyfill, Kristen A. Clarkson, Edwin V. Oaks, Robert W. Kaminski

**Affiliations:** 1 Department of Diarrheal Disease Research, Bacterial Diseases Branch, Walter Reed Army Institute of Research, Silver Spring, Silver Spring, MD 20910, USA; kevin.r.turbyfill.civ@mail.mil (K.R.T.); kristen.a.clarkson.civ@mail.mil (K.A.C.); 2 Patuxent Research and Consulting Group, Gambrills, Gambrills, MD 21054, USA; evoaks@comcast.net

**Keywords:** Invaplex, *Shigella*, vaccine, immunogenicity, adjuvant, antibody

## Abstract

The *Shigella* invasin complex or Invaplex vaccine is a unique subunit approach to generate a protective immune response. Invaplex is a large, macromolecular complex consisting of the major *Shigella* antigens: lipopolysaccharide (LPS) and the invasion plasmid antigen (Ipa) proteins B and C. Over the past several decades, the vaccine has progressed from initial observations through pre-clinical studies to cGMP manufacture and clinical evaluations. The Invaplex product maintains unique biological properties associated with the invasiveness of virulent shigellae and also presents both serotype-specific epitopes, as well as highly conserved invasin protein epitopes, to the immunized host. The vaccine product has evolved from a native product isolated from wild-type shigellae (native Invaplex) to a more defined vaccine produced from purified LPS and recombinant IpaB and IpaC (artificial Invaplex). Each successive “generation” of the vaccine is derived from earlier versions, resulting in improved immunogenicity, homogeneity and effectiveness. The current vaccine, detoxified artificial Invaplex (Invaplex_AR-Detox_), was developed for parenteral administration by incorporating LPS with under-acylated lipid A. Invaplex_AR-Detox_ has demonstrated an excellent safety and immunogenicity profile in initial clinical studies and is advancing toward evaluations in the target populations of children and travelers to endemic countries.

## 1. Introduction

**Figure 1:**
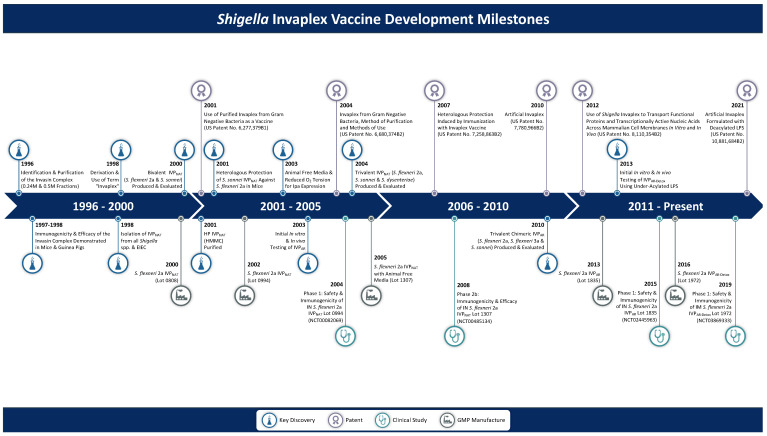
Milestones of Invaplex vaccine development: timeline of key discoveries, patents, cGMP manufactures, and clinical studies associated with the development and evolution of the Invaplex product.

The development and evolution of the *Shigella* invasin complex (Invaplex) family of products has occurred over the past 25-plus years (Figure 1) and represents stepwise, progressive discoveries and improvements that often reflect the current state of knowledge on *Shigella* proteins, lipopolysaccharide (LPS), and immunology. The progress not only involves research on the unique properties of the invasin complex but also critical improvements in product manufacturing, assay development and necessary adjustments to satisfy current regulatory standards and potential commercial development. The Invaplex product today (called artificial detoxified Invaplex or Invaplex_AR-Detox_) represents the latest improvement and is a candidate vaccine that stimulates pronounced systemic and mucosal immune responses in humans that most reflect the immunity observed after natural infection.

## 2. *Shigella* Antigens

After natural infection, the human host responds to several antigens of the *Shigella* pathogen. The most well characterized antigen is LPS, more specifically the O-specific polysaccharide (O-SP) of the LPS, which is responsible for serotype-specific immunity and protective immunity [1,2]. Conjugate vaccines using O-SP coupled to recombinant *Pseudomonas aeruginosa* exoprotein A protected immunized individuals against future disease in studies conducted in the early 1990s [3,4]. Although the O-SP conjugate vaccine approach was very promising and is still being pursued today [5,6,7], it was becoming clear in the late 1980s and early 1990s that a pronounced immune response to the virulence-specific proteins (primarily invasion plasmid antigen (Ipa) proteins IpaB, IpaC and IpaD) also occurred in humans after infection [8]. At the time, the only vaccines capable of stimulating an immune response to both LPS and the Ipa proteins were the live, attenuated vaccines, although the responses to the Ipa proteins were only secondarily evaluated [9,10,11]. More recent CHIM data have complemented field studies and pre-clinical animal studies to make a strong case for immune response to the Ipa proteins playing an important role in protection against shigellosis [12,13,14,15,16].

## 3. Native Invaplex (Invaplex_NAT_)

### 3.1. Discovery of Invaplex

In the mid-1990s, significant advances were underway in the mechanisms by which the Ipa proteins interacted with host cells. Initial reports described the controlled excretion/release of the Ipa proteins as either IpaB/IpaC or IpaB/IpaC/IpaD complexes and established the foundation of the Type III secretion system (TTSS) [17,18,19]. Coupled with this early research on the biomechanics of the Ipa proteins’ secretion and function were the first observations that IpaB, IpaC and IpaD were prominent, conserved antigens recognized by most *Shigella*-infected individuals [8]. Although antigen-specific assays for the Ipa proteins would come later, it was clear that the infected host responded vigorously to virulence-specific antigens found in the water extracts of virulent *Shigella* [8,14,20,21]. It was unclear what role, if any, the host immune response to the highly conserved Ipa proteins had in protection against disease, since dogma clearly identified LPS as the antigen responsible for protective, serotype-specific immunity [1,2,3,22]. Even so, one hypothetical outcome of the response to the conserved Ipa proteins would be the potential for broad protection against all *Shigella* serotypes and enteroinvasive *Escherichia coli* (EIEC). Researchers at the Walter Reed Army Institute of Research (WRAIR) investigated methods to identify and measure the host response to protein antigens. Enzyme-linked immunosorbent assays (ELISA) that could measure the virulence-specific immune response utilized water extracts from wild-type shigellae, which contained the Ipa proteins, VirG (IcsA), LPS and several other undefined proteins [8]. To prepare more specific reagents, attempts to isolate the native Ipa proteins or Ipa complexes from the water extract by various chromatographic methods always resulted in IpaB and IpaC-containing fractions that copurified with LPS [23]. Although the LPS was initially viewed as a “contaminant”, an alternative explanation was that a naturally occurring complex of Ipa proteins and LPS had been isolated. Ultimately, the isolated invasion protein–LPS complex became known as Invaplex and more specifically as native Invaplex (Invaplex_NAT_), the first generation of Invaplex isolated from virulent wild-type shigellae using ion-exchange chromatography [24].

Invaplex_NAT_ could be isolated in two anion-exchange fractions called Invaplex_NAT_ 24 (0.24 M sodium chloride (NaCl) step) and Invaplex_NAT_ 50 (0.50 M NaCl step) [24]. Each Invaplex preparation contained the major *Shigella* antigens IpaB, IpaC, IpaD and LPS and was the formulation for a possible vaccine. Other proteins were also found in Invaplex_NAT_ 24 and Invaplex_NAT_ 50, but neither contained any major outer membrane proteins. The universality of Invaplex_NAT_ was demonstrated by isolating complexes from all *Shigella* species and EIEC [25], which were immunogenic and provided homologous protection in mice and guinea pigs after intranasal immunization [24,25]. Subsequent evaluation of bivalent (*S. flexneri* 2a and *S. sonnei*) and trivalent (*S. flexneri* 2a, *S. dysenteriae* 1 and *S. sonnei*) iterations of the Invaplex_NAT_ product were also successful against homologous challenges [26,27].

### 3.2. Biological Properties of Invaplex_NAT_

The innate *Shigella* virulence attributes of inducing phagocytosis by the host cells and subsequent escape of the bacterium from the phagosome are dependent on functional Ipa proteins [28]. Maintenance of Ipa protein functionality in Invaplex_NAT_ was evaluated in a series of in vitro studies on Invaplex_NAT_ and its ability to interact with non-phagocytic eukaryotic cells. After a brief incubation with host cells Invaplex_NAT_ rapidly decorated the host cell surface, followed by an induced, actin-dependent endocytosis similar to the process utilized by wild-type shigellae. Internalized Invaplex_NAT_ migrated through the retrograde endosomal pathway (early endosome to late endosome to Golgi apparatus) and was ultimately localized free in the cytoplasm [29]. The uptake of Invaplex could be inhibited with antibodies specific for IpaB and IpaC, but antibodies to LPS had no effect on the uptake event. The property of inducing phagocytosis by host cells is considered an essential biological property of Invaplex found in all generations of the product (Table 1) and may be a possible mechanism by which LPS and the Ipa proteins are presented to the host immune system via antigen-presenting cells. In fact, separate studies exploited the native biological activity of Invaplex to deliver and enhance the immune response to otherwise non-immunogenic antigens in which Invaplex_NAT_ was serving as an adjuvant and/or transporter of heterologous, functional cargos (proteins, nucleic acids) into host cells [12,13,15,29,30,31,32]. Furthermore, maintenance of the biological activity suggests that a native structure similar to that found in wild-type shigellae is present in the native complex, making it not only a good vaccine candidate but also a good antigen for use in immunological assays to measure responses to the native invasin complex.

### 3.3. Intranasal Delivery of Invaplex without an Adjuvant

One of the striking features after infection with *Shigella* spp. is the dominant immune response to IpaB, IpaC and IpaD, with a large proportion of infected individuals responding to IpaB and IpaC and a lower proportion responding to IpaD [33,34]. This strong response to the Ipa proteins likely reflects their inherent immunogenicity and critical interaction with the host. Although other *Shigella* proteins are recognized by the immune system, no other antigens stimulate such a dominant, virulence-specific response post-infection. 

Unfortunately, it was unclear how to immunize and stimulate a good mucosal immune response in the intestine other than by oral delivery of live or killed whole bacteria. For subunit vaccines and some inactivated whole-cell vaccines, the use of a strong mucosal adjuvant, such as mutant *E. coli* heat-labile toxin [35], was often necessary to drive a mucosal response. In contrast, immunization in the nasal cavity and exploitation of the “common mucosal pathway” to stimulate migration of mature lymphocytes and production of secretory IgA at distant sites [36,37], such as the intestinal tract, would provide a simple way to stimulate intestinal immunity [38,39]. Utilization of the non-injectable, intranasal route would alleviate many of the clinical concerns associated with administering the wild-type LPS (endotoxin) found in Invaplex_NAT_ and would overcome many limitations of oral immunization with a subunit vaccine, such as antigen quantity, the need for an adjuvant and stability upon delivery into the gastrointestinal tract. 

Pre-clinical experiments clearly demonstrated that animals intranasally immunized with Invaplex_NAT_ produced antibodies to IpaB, IpaC and LPS, suggesting that Invaplex_NAT_ circumvented the poor immunogenicity of LPS delivered alone [40,41]. The intranasal route was used for all animal and human immunizations with Invaplex_NAT_. Pipets were used for all animal studies, but evaluation of different nasal delivery devices was required for humans. Over the course of clinical development, three devices were used for human intranasal immunization: a micropipette, the Dolphin device (Valois of America, Inc., Congers, NY, USA) and the VaxINator™ device (Teleflex, Wayne, PA, USA). Each device was able to deliver the targeted amount of vaccine, with the Dolphin and VaxINator™ devices providing optimal spray distribution to the nasal mucosa. Results from the human studies clearly showed that intranasal immunization led to a significant mucosal immune response in the gut [42,43], which was one of the first direct demonstrations in humans that local intranasal immunizations led to an immune response in the gut. Subsequent studies with the Invaplex vaccine would show that circulating immune lymphocytes carrying the gut mucosal homing marker (α4β7 + B cells) were prominent after intranasal immunization (Invaplex_AR_) and after parenteral immunization (Invaplex_AR-Detox_) (see below).

### 3.4. Immunogenicity of Invaplex_NAT_: Assay Development and Results

Measurement of the specific immune response to the novel Invaplex vaccine has evolved from using traditional ELISAs and other antibody-based assays, such as antibody-secreting cell (ASC) assays to the current use of antigen-specific assays, along with functional assays like the serum bactericidal assay [44,45,46]. The antigens used in early studies consisted of purified LPS to detect serotype-specific responses, the water extract preparations from virulent (Vir+) and avirulent (Vir−) *Shigella* [24] and, later, the Invaplex_NAT_ product. Purified IpaB and IpaC were prominently used in more recent studies once they became readily available and were used to measure a response that would potentially react with all *Shigella* species and EIEC. One antigen frequently used was Invaplex_NAT_ prepared from the same *Shigella* serotype used to produce the vaccine. Studies using sera from animals or humans immunized with the live attenuated vaccine *S. flexneri* 2a SC602 [9,47] or *S. sonnei* WRSS2 and WRSS3 vaccines [48] or Invaplex_NAT_ [24,42,43] consistently demonstrated that there were more seroconversions measured with Invaplex_NAT_ than with any other antigen. Although not fully understood, Invaplex_NAT_, when used as an immunological assay antigen reagent, seems to maintain a native conformation not found in purified LPS, IpaB or IpaC [32]. For this reason, Invaplex_NAT_ has been used as an ELISA antigen to measure the immunogenicity of all Invaplex products. 

The potent immunogenicity of Invaplex_NAT_ after intranasal immunization minimized any need for a mucosal adjuvant. Dosing studies in mice and guinea pigs clearly showed that three biweekly intranasal immunizations of relatively low doses were superior to a two-dose regimen for stimulating a strong, protective immune response. If higher dose amounts were used, a two-dose schedule could be used. In mice, the dose amount ranged from 1 µg to 25 µg, with 5 µg used routinely; in guinea pigs, the routinely used dose amount was 25 µg. The three-dose schedule has been maintained for all Invaplex products, although more recent efforts using parenteral immunizations have spaced out the time schedule to three-week intervals between doses.

After immunization with *S. flexneri* 2a Invaplex_NAT_ 24 or 50, small animals responded with pronounced serum IgA and IgG specific to LPS and the Vir+ water extract [24]. Western blot analysis of guinea pig immune sera indicated that IpaB and IpaC were dominant immunogens in Invaplex_NAT_ 24- and 50-immunized animals, but antibodies to several other non-virulence-specific proteins were found in animals immunized with Invaplex_NAT_ 50. In both guinea pigs and mice immunized with either Invaplex_NAT_ 24 or 50, significant protection was observed upon challenge with homologous, virulent shigellae [24]. Similar immune responses were observed with Invaplex_NAT_ prepared from all *Shigella* species and EIEC. The one notable difference was for *S. sonnei* Invaplex_NAT_ 24, which had much lower quantities of IpaB and undetectable LPS and was not protective in mice or guinea pigs [31,32,49]. These results suggested that either IpaB or LPS or both antigens were crucial for protection in these models. The *S. sonnei* Invaplex_NAT_ 50 product stimulated antibodies to LPS, IpaB, IpaC and other proteins and was fully protective.

### 3.5. Heterologous Immunity Induced by Invaplex

One of the unique features of the Invaplex vaccine products is the ability to deliver, in controlled amounts, a combination of the serotype-specific LPS antigens with highly conserved IpaB and IpaC proteins. The sequence identity between the IpaB and IpaC proteins of different *Shigella* spp. and EIEC is greater than 98% [50,51]. Studies with epitope-defined monoclonal antibodies have demonstrated that several epitopes for IpaB and IpaC are conserved on Ipa proteins from all *Shigella* spp. [16,51]. The high degree of sequence and epitope conservation suggests that a vaccine containing IpaB and IpaC, such as Invaplex, has the potential to stimulate immunity to multiple *Shigella* spp. Using Invaplex_NAT_ 50 prepared from *S. sonnei*, it was possible to protect mice from a lethal challenge with not only the homologous *S. sonnei* but also the heterologous *S. flexneri* 2a [49,52]. The level of protection was highly significant against both challenge organisms. None of the animals immunized with the *S. sonnei* Invaplex_NAT_ 50 produced antibodies to *S. flexneri* 2a LPS, but antibodies to several cross-reactive proteins, including IpaB, IpaC, elongation factor G (84 kDa) and DnaK (72 kDa), were produced after immunization with Invaplex_NAT_ 50 [49,52]. 

The demonstration that cross protection against a heterologous *Shigella* species was possible in the mouse pulmonary model challenged the long-standing concept in humans that LPS was the key protective antigen and that immunity was serotype-specific. Once Invaplex_AR_ was developed, along with its associated purified components, it was demonstrated that heterologous immunity was maintained with a vaccine consisting of only LPS, IpaB and IpaC [53]. Studies in other labs have also demonstrated heterologous immunity in mice with purified IpaB and IpaD and an IpaB/IpaD fusion protein [54,55,56]. However, the broad-based protection identified in the mouse model has not been replicated by this team in the guinea pig model [57]. The mechanism by which conserved protein targeted immunity is involved in protection in mice is unknown, and furthermore, it is not clear what role, if any, it has in humans exposed to shigellae. Even so, as the immune response to the Ipa proteins is dominant post-infection in humans, it is likely the anti-Ipa immune responses are key components of protective immunity [33] and would enhance any vaccine’s effectiveness against the entire *Shigella* genus. As clinical trials progress, investigations on cross-protective immunity will be a key component of the studies to demonstrate a role of anti-Ipa protein immune responses [34].

### 3.6. cGMP Manufacture of Invaplex_NAT_

The encouraging immunogenicity, protection and lack of noticeable side effects in immunized animals led to the initial stages of clinical development including process development for transition to cGMP; development of lot-release criteria that reflected the quality, reproducibility and identity of the product; and adjustments to more readily comply with regulatory guidelines. The first cGMP pilot-scale (30 L) lot of *S. flexneri* 2a Invaplex_NAT_ 50 (Lot 0808) was produced at the WRAIR Pilot Bioproduction Facility (PBF) and released in 2002. *S. flexneri* 2a Invaplex_NAT_ 50 (Lot 0808) was introduced in the initial investigational new drug (IND) application submitted to the US Food and Drug Administration (FDA) in 2004. In subsequent cGMP lots, improved and more reliable expression of the Ipa proteins was achieved by growing the virulent *S. flexneri* 2a strain 2457T with reduced agitation and aeration during the late stages of fermentation (Lot 0994) [58,59] and growth in a WRAIR-developed medium using non-animal-sourced components to remove the concern of bovine spongiform encephalopathy-causing prions in the final product (Lot 1307). Lot 0994 which was used in the first-in-human trials conducted in 2004 [42], and Lot 1307 was used in phase 1 and phase 2b clinical trials from 2007 to 2009 [43,60,61] (see Table 2).

### 3.7. Clinical Evaluation of S. flexneri *2a* Invaplex_NAT_

The novelty of the Invaplex_NAT_ product required new clinical methods and evaluations that permitted the safety and immunogenicity of the *S. flexneri* 2a Invaplex_NAT_ to be thoroughly tested in humans (Table 3). Modeled after experiments in small animals, the three-dose, two-week-interval immunization schedule and the immunological assays were maintained. The first two human trials focused on safety and used a gradual dose escalation (10, 50, 240, 480 and 690 µg) delivered with a micropipette (Lot 0994) [42] or the Dolphin intranasal spray device (Lot 1307) [43]. The Invaplex_NAT_ product was well-tolerated after three immunizations, with no serious adverse events (SAE) and the majority of adverse events (AE) reported as mild. Individuals immunized with the Dolphin device had higher immune responses. The mucosal immune response (ASC levels and fecal IgA), particularly with the high dose (690 µg), were of significant magnitude and frequency to warrant moving the Invaplex_NAT_ vaccine product to a phase 2b inpatient challenge study. An expanded phase 1/2b study was conducted with Lot 1307 in 2008. Unlike the initial phase 1 study with Lot 1307, the second study using only the 690 µg dose amount did not achieve the magnitude or frequency of positive immune responses seen in the earlier studies, and upon challenge, the subjects were not protected, although the challenge study was compromised by low volunteer numbers and a suboptimal level of disease in the unvaccinated controls [60].

Analysis of all clinical study results with Invaplex_NAT_ suggested that a more robust immune response to the three key antigen components (LPS, IpaB and IpaC) would be required to achieve immune responses comparable to natural infection and vaccine efficacy. It was likely that the quantity of specific antigens delivered by the intranasal route in Invaplex_NAT_ needed to be increased to improve the magnitude and frequency of positive immune responses. Furthermore, comments from the FDA clearly signaled that a more defined product would be needed as the vaccine approach matured.

**Table 3:** Clinical evaluations of *Shigella* Invaplex vaccine products.

Study Title	Phase	ClinicalTrials.gov Identifier	Study Start Date	Product (cGMP Lot No.)	Reference
Invaplex 50 Vaccine Dose-Ranging	1	NCT00082069	April 2004	*S. flexneri* 2a Invaplex_NAT_ (0994)	[42]
*Shigella flexneri* 2a Invaplex 50 Vaccine Dose Finding and Assessment of Protection	1	NCT00485134	August 2007	*S. flexneri* 2a Invaplex_NAT_ (0994 and 1307)	[7]
	2b ^1^		January 2008	*S. flexneri* 2a Invaplex_NAT_ (1307)	[60]
	2b		November 2008	*S. flexneri* 2a Invaplex_NAT_ (1307)	
Safety and Immunogenicity of Artificial Invaplex (*Shigella flexneri* 2a Invaplex_AR_) Administered Intranasally to Healthy, Adult Volunteers	1	NCT02445963	October 2015	*S. flexneri* 2a Invaplex_AR_ (1835)	[63]
A Phase 1 Double-blind, Placebo-controlled, Dose Escalating Study of Intramuscular Detoxified *Shigella flexneri* 2a Artificial Invasin Complex (Invaplex_AR-Detox_) Vaccine	1	NCT03869333	March 2019	*S. flexneri* 2a Invaplex_AR-Detox_ (1972)	[64]

^1^ A phase 2b (safety, immunogenicity and efficacy) study was started in January 2008. The study plan included an outpatient immunization phase (WRAIR Clinical Trials Center; Silver Spring, MD, USA), followed by an inpatient challenge phase (Johns Hopkins Bayview; Baltimore, MD, USA). Due to insufficient recruitment for the inpatient phase, the study did not progress to the study challenge phase.

### 3.8. Transition to an Invaplex with Greater Effectiveness

During the process of development and cGMP manufacture of *S. flexneri* 2a Invaplex_NAT_, several observations led to an alternative development plan for the product. It was clear that the Invaplex_NAT_ product had many uncharacterized proteins in the final product, although the immune response stimulated by Invaplex_NAT_ was primarily against IpaB, IpaC and LPS. In addition, the yields of the final Invaplex_NAT_ product would require significant improvement for advanced, larger-scale clinical studies. Further characterization of the Invaplex_NAT_ product focused on the identification and isolation of the “active” component of Invaplex_NAT_. Using size-exclusion chromatography (SEC-FPLC), a high-molecular-mass complex (HMMC) of 1–2 MDa containing only IpaB, IpaC and LPS was isolated from both *S. flexneri* Invaplex_NAT_ 24 and 50 that stimulated strong immune responses to the three antigens and solid protection in mice and guinea pigs. Other SEC-FPLC fractions containing only Ipa proteins and/or other proteins with minimal or no LPS were either not protective or only partially protective [49]. Additionally, the absence of an HMMC in *S. sonnei* Invaplex_NAT_ 24 coinciding with the previously noted lack of IpaB and LPS in *S. sonnei* Invaplex_NAT_ 24 and its lack of protection in small animals suggested that the HMMC is the effector of Invaplex potency and efficacy. The ability to isolate the active component (highly purified Invaplex or HP Invaplex) of Invaplex_NAT_ consisting of only IpaB, IpaC and LPS in an HMMC provided the foundation for the next generation of Invaplex (called artificial Invaplex or Invaplex_AR_) in which purified components were assembled into an HMMC.

## 4. Artificial Invaplex (Invaplex_AR_)

### 4.1. Development of Invaplex_AR_

The isolation of the HP Invaplex established the approximate molar ratio of 8 IpaC:1 IpaB in the native complex and a weight ratio of 0.56 for LPS/total protein. Although the exact origin and mechanism by which Invaplex_NAT_ is formed was not known, assembly experiments to produce Invaplex_AR_ followed, in general, the process by which the water extraction was performed to produce Invaplex_NAT_. The assembly process of Invaplex_AR_ required large quantities of purified IpaB, IpaC and LPS (Table 1). Existing methods to purify each antigen were available but, for the most part, yielded low quantities of the purified component. Therefore, development of improved purification methods for each component were necessary. 

The Westphal LPS purification procedure yields a highly pure LPS with representation of long-chained O-SP and minimal protein contamination (1–2% protein) [65]. By increasing the number of extractions, the yield was significantly improved to produce gram quantities of LPS for each kg of cell paste. The modified procedure was applicable to all *Shigella* species with minor changes and has provided large quantities of LPS not only for Invaplex_AR_ studies but for use as a key immunological reagent for vaccine evaluations used by WRAIR and global collaborators. The improved procedure was transitioned to cGMP manufacture and has been successfully used to produce large quantities of *S. flexneri* 2a LPS. 

Expression of full-size IpaB and IpaC in heterologous hosts such as *E. coli* was initially described in the mid-1990s [66]. The early recombinants produced low yields of the polyhistidine-tagged protein, and the purified proteins were often characterized by poor solubility, largely due to their inherent hydrophobicity [66,67,68]. Using coexpression of IpaB or IpaC with their cognate chaperone IpgC [68] and maintaining solubility of the purified proteins in mild detergents, working quantities of the proteins were produced, allowing research-scale experimentation on structure and function of the proteins [69,70,71,72]. 

**Figure 2:**
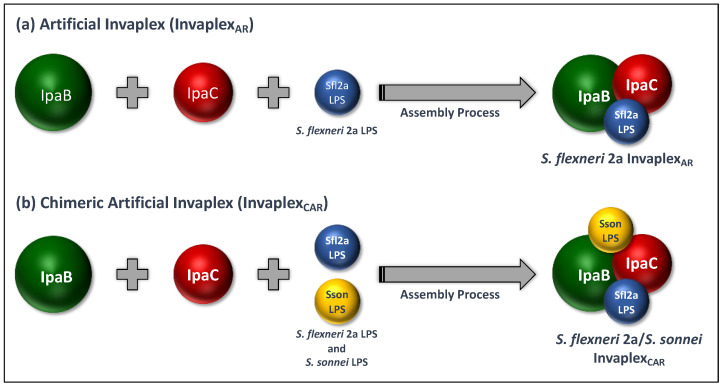
Assembly of artificial Invaplex and chimeric artificial Invaplex: (**a**) artificial Invaplex is assembled in a self-assembly reaction between IpaB, IpaC and LPS in a controlled manner that facilitates customization and quantities of the key immunogens contained within the complex; (**b**) multivalent Invaplex vaccines can be produced using admixtures of artificial Invaplex specific for different serotypes or manufactured as a “chimera” with LPS from multiple *Shigella* serotypes (example shown is a bivalent, chimeric Invaplex) with the complex containing IpaB, IpaC and LPS from more than one serotype.

Initial studies attempting to produce an Invaplex_AR_ used purified LPS from *S. flexneri* 2a and purified IpaB and his-tagged IpaC produced at WRAIR from recombinants kindly provided by the Picking lab. The first artificial Invaplex was produced in 2003 (Figure 2A) with an IpaC:IpaB molar ratio of approximately 5, contained about 1.2 mg LPS/mg protein and had an approximate mass of 1 MDa. The Invaplex_AR_ was soluble, without any added detergent, and was stable frozen or at room temperature (Table 2). Mice or guinea pigs immunized with Invaplex_AR_ had superior immune responses to IpaB, IpaC and LPS as compared to Invaplex_NAT_; Invaplex_AR_ induced solid protection in both animal models [53]. This initial research successfully produced Invaplex_AR_ products for *S. flexneri* 1a, *S. flexneri* 2a, *S. sonnei* and *S. dysenteriae* 1, as well as a construct containing LPS of both *S. flexneri* 2a and *S. sonnei*. The Invaplex product containing LPS from two *Shigella* serotypes complexed with IpaB and IpaC was termed chimeric Invaplex_AR_ or Invaplex_CAR_ (Figure 2B). Although the success of the prototype Invaplex_AR_ was very promising, it was clear that significant improvements in production/purification were necessary prior to cGMP manufacture and transition to clinical evaluation. Critical improvements in the systems used to produce the recombinant IpaB and IpaC were necessary. By using recombinant expression systems with higher plasmid copy numbers, antibiotic selection markers compatible with clinical studies and purification strategies that did not depend on affinity-tagged IpaB or IpaC, significantly increased yields of the target proteins became possible.

Although the prototype Invaplex_AR_ was protective in mice and guinea pigs, studies on the Invaplex_AR_ produced with non-his-tagged IpaC and IpaB sought to maximize immune responses to all antigens, with emphasis on LPS. Enhanced immune responses to all Invaplex antigens were considered key to the vaccine’s effectiveness in humans. By modifying the Invaplex_AR_ assembly and purification protocols to include different amounts and ratios of each reactant/antigen, it was possible to produce an Invaplex_AR_ that contained greater quantities of LPS, achieved an IpaC:IpaB ratio approaching the ratio identified with HP Invaplex, had a high molecular mass and, most importantly, stimulated a solid immune response to all three components. The modified assembly strategy has also been successfully used to produce Invaplex_AR_ for *S. flexneri* 3a, *S. flexneri* 6 and *S. sonnei* (Table 4), which are the leading target serotypes for a multivalent *Shigella* vaccine [73,74]. 

### 4.2. cGMP Manufacture of Invaplex_AR_

The first Invaplex_AR_ cGMP lot was manufactured at the WRAIR PBF in 2013 (Lot 1835). Prior to the assembly phase of Invaplex_AR_, cGMP lots of purified *S. flexneri* 2a LPS, IpaB and IpaC were manufactured using protocols and master cell banks developed at WRAIR. Gram quantities of each antigen were produced and stored frozen. The cGMP lots of IpaB, IpaC and *S. flexneri* 2a LPS were used to assemble the final Invaplex_AR_ product. Lot 1835 characteristics are outlined in Table 2, which compares multiple generations of cGMP Invaplex lots. Noteworthy characteristics include purity (well-defined composition), immunogenicity, protective efficacy and lack of adverse side effects in small animals.

### 4.3. Clinical Evaluation of S. flexneri *2a* Invaplex_AR_

The initial Phase 1 trial for Invaplex_AR_ Lot 1835 was conducted in 2015 (Table 3). Volunteers were immunized intranasally with four dose amounts of Invaplex_AR_ (10, 50, 250 and 500 µg) on days 0, 14 and 28 [75]. The primary goals related to safety and effective delivery of the vaccine with the VaxINator™ nasal spray device were achieved. The vaccine was safe and well tolerated at all doses tested, and no AEs met stopping criteria. The most common AEs were nasal congestion, rhinorrhea and postnasal drip. The AE rates were consistent with prior intranasal immunizations with *S. flexneri* 2a Invaplex_NAT_ [42,43] and resolved within 72 h. Intranasal immunization with ≥50 µg of Invaplex_AR_ induced serum IgG and IgA directed to Invaplex_NAT_ and LPS antigens, with no clear dose response and no significant increase in titer after the third immunization. ASC or antibody in lymphocyte supernatant (ALS) assays detected IgA- and IgG-secreting cells specific to Invaplex_NAT_ after immunization with ≥50 µg of Invaplex_AR_. Antibodies to LPS and Invaplex_NAT_ were also detected in fecal and saliva samples in 38% to 67% of the volunteers. Functional bactericidal serum antibodies were detected in ≥50% of individuals in the 50 µg and 500 µg dose cohorts. Overall, the phase 1 trial with Invaplex_AR_ showed a safety profile consistent with that of previous studies with Invaplex_NAT_ and demonstrated improved immunogenicity to key *Shigella* antigens. Despite these encouraging results, a robust immune response was not present in a high proportion of individuals, suggesting that additional adjustments were necessary.

## 5. Artificial Detoxified Invaplex (Invaplex_AR-Detox_)

Although the safety profile and immunogenicity of Invaplex vaccines delivered intranasally were encouraging, the potential for combining Invaplex with other licensed vaccines and uptake into existing immunization programs suggested that parenteral immunization may be preferable. Therefore, the current generation of Invaplex_AR_ is formulated with LPS containing an under-acylated lipid A moiety (artificial detoxified Invaplex or Invaplex_AR-Detox_) instead of fully-acylated lipid A. Transition to the under-acylated LPS widened the vaccine-associated safety window to permit parenteral administration by reducing but not completely abrogating the danger signal associated with endotoxin, resulting in a highly immunogenic vaccine.

### 5.1. Development and Pre-Clinical Testing of Invaplex_AR-Detox_

The inflammatory activity of lipid A (endotoxicity) is dependent on interactions with the Toll-like receptor (TLR)-4 complex (MD-2, CD14 and TLR-4). The number and position of acyl chains contribute to binding between lipid A and the TLR-4 complex, with the hexa-acylated lipid A moiety having the highest biological activity. Both penta- and tetraacylated lipid A have been shown to have reduced or attenuated endotoxic activity [76]. Therefore, studies were conducted to evaluate the inflammatory potential of LPS isolated from mutant *Shigella* strains with single and double deletions of the *msb*B gene, which is a late acyl transferase. Although LPS from the single *msb*B mutants were found to be less proinflammatory as compared to fully acylated lipid A, LPS from the double *msb*B mutant strain (WR30) was utilized for downstream development [77]. Furthermore, when pilot batches of Invaplex_AR-Detox_ assembled with *S. flexneri* 2a LPS from the various *msb*B *Shigella* mutants were utilized in monocyte activation experiments, the level of proinflammatory cytokines (TNF-alpha and IL-6) released was significantly lower as compared to the cytokines released after monocyte incubation with similar amounts of LPS from the mutated *Shigella* strains, indicating that complexing the LPS with proteins further reduced the proinflammatory potential of the vaccine [78].

### 5.2. Pre-Clinical Studies with Invaplex_AR-Detox_

The impact of Invaplex_AR-Detox_’s exceptional immunogenicity has been demonstrated in several animal models. Guinea pigs immunized parenterally or intranasally with *S. flexneri* 2a Invaplex_AR-Detox_ were significantly protected in the Sereny model, whereas no animals in the control group were protected. Using a refinement to the guinea pig rectocolitis challenge model [79], animals immunized with Invaplex_AR-Detox_ were also significantly protected from disease (protective efficacy = 67%; *p*-value = 0.008). In this experiment, myeloperoxidase (MPO) concentrations, an indicator of gut inflammation, were determined in stool samples collected pre-challenge and 1, 2 and 3 days post-challenge. High levels of MPO (≥1500 ng/mL), indicating inflammation in the gut, were detected post-challenge in stool of the saline-treated animals; however, MPO was reduced to negligible levels (≤250 ng/mL) in the animals vaccinated intramuscularly with Invaplex_AR-Detox_. Collectively, these studies demonstrate that Invaplex_AR-Detox_ was protective and has the capacity to significantly reduce intestinal inflammation, potentially offering added benefits to children in endemic settings because elevated MPO levels have been considered a marker for increased risk for developing stunting and gut enteropathy [80]. The encouraging results from the in vitro and in vivo assessments of *S. flexneri* 2a Invaplex_AR-Detox_ led to the cGMP manufacture of the product at the WRAIR PBF, which was completed in 2015. A double *msb*B mutant of wild-type *S. flexneri* serotype 2a strain 2457T WR30 in which both *msb*B1 and *msb*B2 were deleted was used in combination with the same cGMP manufactured lots of IpaB and IpaC used to manufacture Invaplex_AR_ (Lot 1835), as described previously (Table 2).

### 5.3. Clinical Evaluation of Invaplex_AR-Detox_

cGMP Invaplex_AR-Detox_ Lot 1972 was evaluated in a first-in-human study during 2019 (Table 3) in which the vaccine was delivered to three cohorts with intramuscular doses of 2.5, 10 and 25 µg delivered on days 0, 21 and 42. The vaccine was well tolerated, with no SAEs and more than 90% of the recorded AEs scored as mild. AEs were dose-dependent and reduced with frequency of administration. The most frequent AEs were pain and tenderness at the injection site and occurred most frequently after the first injection. All three dose levels of Invaplex_AR-Detox_ were highly immunogenic, inducing strong serum antibody responses to all *Shigella* antigens (LPS, IpaB and IpaC) contained within the vaccine. The magnitude of the LPS-specific serum IgG responses were comparable to or exceeded antibody levels observed after intranasal immunization with Invaplex_NAT_ [42,43] and Invaplex_AR_ [75], infection with *S. flexneri* 2a or after immunization with other vaccine candidates [6]. In addition to robust systemic responses, the Invaplex_AR-Detox_ vaccine elicited significant mucosal IgG and IgA responses, with an average of 93% of vaccinated subjects demonstrating a mucosal antibody response across all dose levels [78]. The Invaplex_AR-Detox_ vaccine also induced robust serum bactericidal antibodies capable of killing *S. flexneri* 2a (the targeted organism) that were comparable to or exceeded those induced after infection with wild-type *S. flexneri* 2a [81]. The high level of functional antibodies induced after vaccination were maintained ≥1.4 years after the primary immunization. More importantly, immunization with Invaplex_AR-Detox_ induced a broad bactericidal antibody response capable of killing other *Shigella* serotypes that also contribute to global morbidity and mortality (*S. flexneri* 3a and 6). These findings may be associated with the fact that the Invaplex_AR-Detox_ used in the study was the first parenteral *Shigella* vaccine that delivered controlled amounts of Ipa proteins, which are broadly conserved and surface-expressed in all *Shigella* serotypes. Invaplex_AR-Detox_ also induced LPS and Ipa protein-specific immunological memory B cell responses, which are key components of a protective immune response after immunization.

## 6. Next Steps and Future Directions

The Invaplex_AR_ and Invaplex_AR-Detox_ vaccines represent the first subunit products to deliver controlled amounts of the Ipa proteins and serotype-specific LPS to induce robust immune responses in humans. The customization afforded by the Invaplex approach greatly facilitates expansion to other *Shigella* serotypes. Moreover, the inherent adjuvant properties of Invaplex also hold the promise of enhancing immune response to codelivered vaccines in the absence of an exogenous adjuvant, paving the way for less expensive combination vaccine approaches.

The excellent safety profile, tolerability and immunogenicity of the Invaplex_AR-Detox_ product warrant further evaluations and development. Several activities on the critical product development pathway will occur in parallel over the coming years. Efforts are underway to cGMP manufacture an *S. sonnei* Invaplex_AR-Detox_ vaccine. The new product will be evaluated alone and in combination with the *S. flexneri* 2a Invaplex_AR-Detox_ vaccine to establish safety and preliminary dosing levels of the multivalent vaccine. Given the cross reactivity of functional antibody responses generated in the phase 1 study with *S. flexneri* 2a Invaplex_AR-Detox_, it may be possible to provide sufficient coverage against the four major *Shigella* serotypes with a bivalent Invaplex_AR-Detox_ product. In parallel, further efforts towards a stable Invaplex_AR-Detox_ formulation that will not be as dependent on the cold chain are underway. It is also anticipated that the Invaplex_AR-Detox_ product will enter age-descending and dose-finding studies in adults, children and infants living in endemic settings to determine immunogenicity in target populations.

## Figures and Tables

**Table 1:** Summary of the Invaplex product family.

Attribute/Property	1st Generation	2nd Generation	3rd Generation
**Name**	Native Invaplex (Invaplex_NAT_)	Artificial Invaplex (Invaplex_AR_)	Detoxified Artificial Invaplex (Invaplex_AR-Detox_)
**Date of Discovery**	1996	2003	2013
**Source**	Isolated from wild-type shigellae	Assembled from recombinant proteins and wild-type LPS	Assembled from recombinant proteins and under-acylated LPS
**Complex Composition**	IpaB IpaC IpaD Wild-type LPS Unidentified proteins	IpaB IpaC Wild-type LPS	IpaB IpaC Under-acylated LPS
**First cGMP Manufacture Date**	2000	2013	2016
**Adjuvant Activity**	✓	✓	✓
**Induces Uptake** **(Non-Phagocytic Cells)**	✓	✓	✓
**Produced for Multiple** ***Shigella* Serotypes**	*S. flexneri* 2a *S. sonnei* *S. dysenteriae* *S. boydii*	*S. flexneri* 2a *S. flexneri* 3a *S. flexneri* 6 *S. flexneri* 1b *S. dysenteriae* *S. sonnei*	*S. flexneri* 2a *S. sonnei*
**Route of Immunization**	Intranasal	Intranasal	Parenteral
**Efficacious in Mice**	✓	✓	✓
**Efficacious in Guinea Pigs**	✓	✓	✓
**Heterologous Protection (Mice)**	✓	✓	Not Tested

**Table 2:** Comparison of cGMP *S. flexneri* 2a Invaplex products.

Analysis		Invaplex_NAT_ (cGMP Lot 1307)	Invaplex_AR_ (cGMP Lot 1835)	Invaplex_AR-Detox_ (cGMP Lot 1972)
**Endotoxin (EU/mL) ^1^**		0.6 × 10^7^	5.3 × 10^7^	4.6 × 10^7^
**Protein Concentration (mg/mL) ^2^**		3.5	2.9	2.8
**IpaC: IpaB Ratio ^3^**		1.5:1	6.8:1	5.4:1
**LPS: Total Protein Ratio (EU/mg)**		0.17 × 10^7^	1.8 × 10^7^	1.6 × 10^7^
**SEC Retention Time (mins) ^4^**		17.4 Multiple Peaks	15.7 Single Peak	16.2 Single Peak
**Size, D*_H_* (nm ± standard deviation) ^5^**		10 ± 3	24 ± 3	24 ± 4
**Immunogenicity ^6^**		2036 (100%) *p* < 0.001	8146 (100%) *p* < 0.0001	146,295 (100%) *p* < 0.0001
**Protective Efficacy ^7^**		83% *p* < 0.001	100% *p* < 0.0001	75% *p* = 0.0003
**GLP Toxicology**		No significant histopathology in mice after IN immunization	No significant histopathology in mice after IN immunization	No significant adverse findings in rabbits after IM immunization
**Pyrogenicity**		Negative in rabbits (IN immunization)	Negative in rabbits (IN immunization)	Monocyte Activation Test—PASS
**Stability at −80 °C (no loss of antigen content or immunogenicity)**		≥3 years	≥5 years	≥5 years
**Stage of Clinical Development**		Phase 1: Safe and immunogenic P2b: Less immunogenic and not protective	Phase 1: Safe and immunogenic	Phase 1: Safe and highly immunogenic
**Lowest Immunogenic Dose for ≥50% Seroconversion in Humans**	**Anti-Invaplex_NAT_** **IgG**	690 µg	50 µg	2.5 µg
	**Anti-LPS IgG**	690 µg	250 µg	2.5 µg

^1^ Endotoxin was measured by Limulus amebocyte lysate assay. ^2^ Final total protein concentration was determined by Pierce BCA total protein assay against a BSA standard curve. ^3^ The IpaC:IpaB ratio was determined by quantitative ELISA for Lot 1307. For Lots 1835 and 1972, IpaC:IpaB ratios were determined by scanning Coomassie-stained SDS-PAGE gels using a Bio-Rad GS900 calibrated flatbed scanner, and the pixel densitometric ratios were calculated using Bio-Rad Image Lab software. ^4^ Size-exclusion chromatography (SEC-HPLC) was performed using a TSK-GEL G5000PWXL 7.8 mm × 30 cm column (TOSOH Bioscience) with a 10 µm particle size and an exclusion limit of 1 × 10^7^ Daltons connected to a Dionex UltiMate™ 3000 UHPLC with a diode array detector reading at a 215 nm wavelength. ^5^ Dynamic light scattering (DLS) measurements of hydrodynamic diameter (DH) in nm were performed using a Malvern zetasizer µV DLS/SLS detector. ^6^ Guinea pigs were immunized intranasally or intramuscularly three times at two-week intervals (days 0, 14, 28) with either 25 µg of the cGMP Invaplex product or saline (not shown). Blood was collected at baseline (day 0) and two weeks after the last immunization (day 42) to determine vaccine-induced serum IgG responses by ELISA. Outlined above are Invaplex_NAT_-specific geometric mean serum IgG ELISA endpoint titers on day 42 with the percentage of responders in parentheses (defined as a ≥4-fold rise over baseline). *p*-values were determined by two-way ANOVA with Bonferroni post hoc test comparing the immunized group to the saline control group. ^7^ Guinea pigs were ocularly challenged 3 weeks after the last immunization with ~2 × 10^8^ cfu/eye of *S. flexneri* 2a, 2457T. The eyes of each animal were monitored daily for inflammation and keratoconjunctivitis. Disease was graded as previously described [62], with scores ≥ 2 considered positive for disease or unprotected. Results 5 days post-challenge were used to determine protective efficacy, calculated as ((% disease in control animals—% disease in vaccine group)/% disease in control animals) × 100. *p*-values were determined by Fisher’s exact test, comparing the immunized group to the saline control group.

**Table 4:** Invaplex_AR_ products for the four most globally prevalent *Shigella* serotypes.

*Shigella* Species	Endotoxin (×10^6^ EU/mL) ^1^	IpaC:IpaB Ratio ^2^	SEC-HPLC Retention Time (min) ^3^	DLS DH (nm) ^4^	Immunogenicity (Responses Directed to IpaB, IpaC and LPS) ^5^	Efficacy (*p* Value) ^6^
*S. flexneri* 2a	63	7.8:1	15.6	19.5 ± 7.7	Yes	75% (*p* = 0.0005)
*S. flexneri* 3a	16	3.8:1	16.2	19.5 ± 4.8	Yes	70% (*p* = 0.012)
*S. flexneri* 6	220	3.9:1	16.0	28.2 ± 0.4	Yes	71% (*p* = 0.0213)
*S. sonnei*	5.7	2.6:1	16.1	19.5 ± 4.0	Yes	92% (*p* < 0.0001)

^1^ Endotoxin was measured using the Charles River Laboratories EndoSafe^®^-PTS endotoxin reader and FDA-licensed cartridges with a sensitivity of 0.001 EU/mL. ^2^ The IpaC:IpaB ratio was determined by scanning Coomassie-stained SDS-PAGE gels using a Bio-Rad GS900 calibrated flatbed scanner and the pixel densitometric ratios calculated using Bio-Rad Image Lab v software. ^3^ Size-exclusion chromatography (SEC-HPLC) was performed using a TSK-GEL G5000PWXL 7.8 mm × 30 cm column (TOSOH Bioscience) with a 10 µm particle size and an exclusion limit of 1 × 10^7^ Daltons connected to a Dionex UltiMate™ 3000 UHPLC with a diode array detector reading at a 215 nm wavelength. ^4^ Dynamic light scattering (DLS) measurements of hydrodynamic diameter (DH) in nanometers were performed using a Malvern zetasizer µV DLS/SLS detector. ^5^ Serum IgG specific for IpaB, IpaC and homologous serotype-specific LPS was determined by ELISA. Responders were defined as ≥4-fold increase in antigen-specific titers over baseline. ^6^ Guinea pigs were ocularly challenged 21 days after the final immunization with *Shigella* spp. (~2 × 10^8^ cfu/eye) homologous to the *Shigella* serotype targeted by the monovalent Invaplex vaccine product. The eyes of each animal were monitored daily for inflammation and keratoconjunctivitis. Disease was graded as previously described [62], with scores ≥ 2 considered positive for disease or unprotected. Results 5 days post-challenge were used to determine protective efficacy, calculated as ((% disease in control animals—% disease in vaccine group)/% disease in control animals) × 100. *p*-values were determined by Fisher’s exact test, comparing the immunized group to the saline control group.

## Data Availability

Not applicable.

## Disclaimer

Some authors (K.R. Turbyfill, K.A. Clarkson, R.W. Kaminski) are employees of the U.S. Government. Material has been reviewed by the Walter Reed Army Institute of Research. There is no objection to its presentation and/or publication. The opinions or assertions contained herein are the private views of the author and are not to be construed as official or as reflecting true views of the Department of the Army, nor the Department of Defense, nor the U.S. Government. The investigators have adhered to the policies for protection of human subjects as prescribed in AR 70–25.

## Copyright Statement

Some authors (K.R. Turbyfill, K.A. Clarkson, R.W. Kaminski) are employees of the U.S. Government. This work was prepared as part of their official duties. Title 17 U.S.C. §105 provides that “Copyright protection under this title is not available for any work of the United States Government.” Title 17 U.S.C. §101 defines a U.S. Government work as a work prepared by a military service member or employee of the U.S. Government as part of that person’s official duties.
